# The Early Functional Abilities (EFA) scale to assess neurological and neurosurgical early rehabilitation patients

**DOI:** 10.1186/s12883-015-0469-z

**Published:** 2015-10-19

**Authors:** Ariane Hankemeier, Jens D. Rollnik

**Affiliations:** Institute for Neurorehabilitation Research (InFo), BDH Clinic Hessisch Oldendorf, Teaching Hospital of Hannover Medical School (MHH), Greitstr. 18-28, 31840 Hessisch Oldendorf, Germany

**Keywords:** EFA, Early functional abilities, Rehabilitation, Neurology, Neurosurgery, Outcome

## Abstract

**Background:**

It is difficult to assess neurological and neurosurgical early rehabilitation patients comprehensively. Available scales focus on activities of daily living (Barthel (BI) and Early Rehabilitation Barthel Index (ERBI)) or wakefulness (Glasgow Coma Scale (GCS), Coma Remission Scale (CRS)) while cognitive items are missing.

**Methods:**

The Early Functional Abilities (EFA) scale comprises 20 items referring to activities of daily living (ADL), wakefulness and cognitive abilities. To evaluate its validity, *n* = 623 early neurological and neurosurgical rehabilitation patients (most of them after ischemic stroke or cerebral bleeding) were assessed on admission using the EFA, ERBI, GCS, CRS and measures of morbidity (co-diagnoses).

**Results:**

The more co-diagnoses the lower EFA sum scores were obtained (Spearman-Rho r_s_ = -0.509, p < 0.001). EFA predicted length of stay (LOS, r_s_ = -0.565, *p* < 0.001) and BI at discharge (r_s_ = 0.571, *p* < 0.001).

**Conclusions:**

The results suggest that EFA is a valid instrument to assess critically ill neurological and neurosurgical early rehabilitation patients. It may be used as a measure of morbidity and a predictor of LOS and outcome. Further studies are strongly encouraged.

## Background

Patients with severe neurological impairment, e.g. after stroke or brain injury, require specialized early rehabilitation following acute hospital treatment [1, 2]. An early initiation of rehabilitation is essential because it may lead to a better neurological outcome [3]. These patients frequently suffer from disorders of consciousness, high morbidity and functional dependence [1, 2, 4]. In addition, many are dependent on intensive care treatment and mechanical ventilation while undergoing neurological early rehabilitation [5] and may be colonized with multi-drug resistant germs [6].

A valid assessment of neurological early rehabilitation patients is difficult. The Barthel Index (BI), for instance, is a measure of independence in activities of daily living (ADL) and even allows length of stay prediction in subsequent rehabilitation [7]. However, there are ceiling effects and change sensitivity of the BI is low when assessing severely impaired neurological early rehabilitation patients [8]. For that reason, an extension of the BI has been developed, the so-called “Early Rehabilitation Barthel Index” (ERBI) [4, 8–10]. The ERBI has even entered the German ICD-10 catalogue [11] and the definition of the early neurological rehabilitation procedure 8-552 in the German DRG system (ERBI ≤ 30 as inclusion criterion) [12]. It includes highly relevant items like tracheostomy, mechanical ventilation or monitoring on a dichotomic scale, but only few studies have proven its validity in neurological early rehabilitation [4]. Further, the ERBI has limitations because it does not allow evaluation of wakefulness and cognitive abilities.

Most other assessments frequently used in early rehabilitation focus on wakefulness/consciousness, like Glasgow Coma Scale (GCS) [13] or Coma Remission Scale (CRS) which is mainly used in Germany [14]. An evidence-based review included 13 more assessment scales for disturbances of consciousness [15]. Among these, the Coma Recovery Scale – Revised (CRS-R) [16] was recommended with minor reservations to assess consciousness. The Sensory Modality Assessment Technique (SMART) [17], Western Neuro Sensory Stimulation Profile (WNSSP) [18], Sensory Stimulation Assessment Measure (SSAM) [19], Wessex Head Injury Matrix (WHIM) [20] and Disorders of Consciousness Scale (DOCS) [21, 22] may be used to assess consciousness with moderate and the Coma/Near-Coma Scale (CNC) [23] with major reservations as far as reliability and diagnostic/prognostic validity are concerned [15].

The German Early Functional Abilities (EFA) scale has been introduced to assess both – ADL and cognitive functions (including wakefulness) of neursosurgical early rehabilitation patients [24, 25]. The EFA scale (Table 1) comprises 20 items in 4 categories (autonomic, oro-facial, sensorimotor and cognitive functions/abilities). Each item is rated on a five-point-scale: 1 = “no function”, 2 = “severe disturbance”, 3 = “moderate disturbance”, 4 = “slight disturbance”, 5 = “normal” [24, 25]. Thus, EFA total scores may range from 20 to 100 [24, 25]. Besides vegetative abilities, inter-rater reliability of the EFA scale was found to be moderate to good for most other items [25]. With respect to its validity, however, there is a considerable lack of evidence.

**Table 1:** Categories and items of the Early Functional Abilities (EFA) scale

Categories	Items	Rating [1–5]
A) Vegetative functions	1) Autonomic stability	1- Instable even at rest, monitoring required
		2 - Stable at rest but requires monitoring at least temporarily
		3 - Stable at rest and during nursing, no monitoring required
		4 - Slightly instable only during rehabilitation therapy
		5 - No marked changes in blood pressure, heart rate or perspiration during nursing or rehabilitation therapy
	2) Wakefulness	1 - Lack of regular sleep-wake cycle, nocturnal agitation, sleepy during the day
		2 - Infrequent nocturnal agitation and sleepy during the day
		3 - Regular sleep-wake-cycle established
		4 - Fatigued after rehabilitation therapy of 10-60 min duration
		5 - No fatigue, even after rehabilitation therapy of more than 60 min
	3) Tolerance to postural changes	1 - Only supine position is tolerated well, lying on one side less than 20 min
		2 - Lying on one side tolerated 20-60 min
		3 - Lying on one side tolerated 60-120 min
		4 - Lying on one side tolerated more than 120 min
		5 - Complete tolerance to postural changes
	4) Excretion functions (continence)	1 - No faecal and urinary continence at all, urinary catheter
		2 - Use of diaper or urine bottle where possible
		3 - Use of toilet-chair where possible, no urinary catheter
		4 - Continence during the day, nocturnal incontinence
		5 - Faecal and urinary continence
	*Category score*	*4 to 20 points*
B) Oro-facial-functions	5) Oro-facial stimulation/oral hygiene	1- No cooperation, no reaction to oro-facial stimulation
		2 - No cooperation, minor reactions upon stimulation
		3 - Partial cooperation (e.g. opening mouth)
		4 - Good cooperation during oral hygiene
		5 - Oral hygiene carried out independently (e.g. toothbrushing)
	6) Swallowing	1 - No/infrequent swallowing of saliva, high danger of aspiration
		2 - Swallowing of saliva improved, still danger of aspiration
		3 - Swallowing of mush possible, drinking still dangerous (aspiration)
		4 - No disturbance of swallowing of food, drinking infrequently disturbed
		5 - No disturbance of eating and drinking
	7) Tongue movements/chewing	1 - No tongue movements, no chewing
		2 - Severely disturbed chewing
		3 - Chewing improved, tongue movements severely disturbed
		4 - Tongue movements improved
		5 - No disturbance of tongue movements or chewing.
	8) Facial expression	1 - No facial expression/reaction
		2 - Some spontaneous facial expression/reaction
		3 - Infrequent spontaneous and voluntary facial expression
		4- Slightly disturbed voluntary facial expression
		5 - Regular facial expression.
	*Category score:*	*4 to 20 points*
C) Sensorimotor abilities	9) Muscle tone	1 - No modulation of muscle tone (spastic or floppy)
		2 - Some modulation of muscle tone may be observed in unaffected limbs
		3 - Improved modulation and holding of muscle tone
		4 - Good modulation and holding of muscle tone in unaffected limbs
		5 - Normal modulation and holding of muscle tone in supine position
	10) Head postural control	1 - No head postural control at all
		2 - Severely disturbed head postural control during rehabilitation therapy
		3 - Infrequently raising the head
		4 - Holding up the head for up to 10 min
		5 - Normal head posture, longer thann 10 min
	11) Trunk postural control/sitting	1 - No sitting at all
		2 - Passive sitting
		3 - Active sitting, infrequentl correction of trunk position
		4 - Active sitting without any help, less than 10 min, still some problems in keeping balance
		5 - Physiological trunk posture; sits without help more than 10 min
	12) Changing position	1 - No voluntary changes of position
		2 - Changes position with help from 1-2 nurses
		3 - Changes position with little help from 1 nurse
		4 - Changes position almost without any help
		5 - Can stand up from a lying position without any help.
	13) Standing	1 - No standing at all.
		2 - Stands (passively) only 5-10 min with help from 2 nurses
		3 - Stands (passively) more than 10 min with help from 2 nurses
		4 - Stands (actively) with help from only 1 nurse
		5 - Stands without help
	14) Voluntary movements	1 - No voluntary movements
		2 - Infrequent voluntary movements (e.g. aversion motions)
		3 - Grasps, but does not let go
		4 - Slight disturbance of grasping and letting
		5 - No disturbance of voluntary movements
	15) Locomotion/mobility in wheelchair	1 - No use of a wheelchair at all
		2 - Passive transport in wheelchair
		3 - Patient has trunk and head postural control in wheelchair
		4 - Active use of wheelchair by the patient or walking some steps
		5 - Independent mobility in wheelchair or ambulation without help
	*Category score:*	*7 to 35 points*
D) Cognitive abilities	16) Tactile stimulation	1 - No response to tactile stimulation
		2 - Nonspecific response to stimulation (e.g. agitation, heart rate or muscle tone changes)
		3 -Voluntary response to stimulation, in particular aversion
		4 - Grasping or other targeted actions
		5 - Adequate reactions to tactile stimulation
	17) Visual stimulation	1 - No response to visual stimulation
		2- Nonspecific response or short eye contact
		3 -Voluntary response to stimulation, eye contact
		4 - Targeted actions, eyes search the environment
		5 - Adequate reactions to visual stimulation
	18) Auditory stimulation	1 - No response to auditory stimulation
		2 - Nonspecific response to stimulation (e.g. agitation, heart rate or muscle tone changes)
		3 - Voluntary response to stimulation, orientation of eye or head movement to the stimulus
		4 - Different reactions to familiar /unfamiliar voices
		5 - Assimilation of acoustic information over longer periods of time.
	19) Communication	1 - None.
		2 - Low-level communication (e.g. expression of discomfort)
		3 - Infrequent adequate responses
		4 - Patient is able to answer with yes/no
		5 - Talking or communicating without problems
	20) Comprehension	1 - None
		2 - Nonspecific reactions (e.g. muscle tone changes)
		3 - Patient is more cooperative, partial comprehension of the situation, apractic/agnostic
		4 - Comprehension improved, no apraxia/agnosia
		5 - No disturbance of activities of daily living.
	*Category score:*	*5 to 25*
	*EFA total score:*	*20 to 100 points*

Compared to other scales which are focusing on consciousness [15], the EFA also allows an assessment of vegetative (e.g. tolerance to postural changes and excretion functions) and oro-facial functions (e.g. oral hygiene, tongue movements and chewing) [24, 25]. Further, it has to be pointed out that neurological and neurosurgical early rehabilitation patients frequently suffer from disturbances of consciousness at early stages but with improving awareness, sensorimotor and cognitive functions are more and more important for the patients` recovery. Therefore, an evaluation of vegetative, oro-facial, sensorimotor and cognitive functions through only one assessment tool is very useful. The EFA allows a monitoring of the patients` progress throughout the whole early rehabilitation process.

In the present study, the EFA scale was used in a cohort of neurological and neurosurgical early rehabilitation patients. The study`s goals were defined as follows:

1.  Examination of concurrent validity
  The study wanted to explore whether it was associated with expected measures of severity and co-morbidity. The Well established assessments (like Barthel index) have been measured concurrently to study its concurrent validity.
2.  Examination of prognostic validity
  Further, the prognostic validity was explored to see whether EFA scores on admission were predictive of later outcomes and length of stay (LOS).

## Methods

The BDH Clinic Hessisch Oldendorf, Germany, is a teaching hospital of Hannover Medical School (MHH). It offers acute hospital treatment (including stroke unit and intensive care treatment), neurological and neurosurgical early rehabilitation, as well as subsequent rehabilitation (e.g. medical-occupational rehabilitation [26]).

Medical records of *n* = 623 early rehabilitation patients admitted to the clinic in 2010 have been carefully reviewed with respect to age, morbidity, length of stay (LOS) and functional independence (ADL) measures. On admission, the following scales/assessments have been applied: ERBI [4], GCS [13], CRS [14] and EFA [24, 25]. In addition, ERBI (including BI) at discharge was used to evaluate outcome. The ERBI consists of the Early Rehabilitation Index (ERI) and BI (ERBI = ERI + BI) [4]. Each item of the ERI (intensive care treatment, mechanical ventilation, confused patient, behavioural disturbances, impairment of communication, dysphagia) is rated on a dichotomic scale [4]. If an item is applicable, it scores with a minus value (-50 or -25 points) [4]. The ERI sum score (-325 to 0 points) is added to the BI (0 to +100 points). Thus, the total ERBI scores range from -325 to +100 [4]. GCS ranges from 3 to 15 [13], CRS from 0 to 24 [14].

BI served as primary outcome variable. According to previous studies, a poor outcome was defined as BI < 50 at discharge [27, 28].

In addition, length of stay (LOS), period of time until first remission signs were observed (remission time in patients with disorders of consciousness, e.g. eye contact), duration of autonomic instability (defined as at least 1 week without any autonomic instability like body temperature, blood pressure or heart rate peaks) and morbidity parameters (number of co-diagnoses, Patient Clinical Complexity Level [2]) have been analyzed. As neurophysiologic measure of wakefulness, EEG rhythms of *n* = 183 patients have also been included in the analysis.

Only those cases have been added to the database with a complete assessment of at least EFA, BI and ERI. The assessors have been nurses (BI, ERI, EFA vegetative functions), physicians (GCS, CRS, review of BI and ERI), speech therapists (EFA oro-facial), physiotherapists (EFA sensorimotor) and occupational therapists (EFA cognitive) who were well experienced in neurological and neurosurgical early rehabilitation.

In the results section, mean values and standard deviations (in brackets) are displayed. In parametric testing (t-tests and ANOVAs with post-hoc LSD-tests), differences were regarded as significant with *p* < 0.05. Correlations were computed using the Spearman-Rho correlation coefficient (r_s_).

Local ethics committee (BDH-Clinic Hessisch Oldendorf) determined that the study was exempt from ethics approval and hence waived approval because the study was a retrospective database analysis, only (no intervention).

## Results

### Patient cohort

Data of *n* = 623 (*n* = 283 female, *n* = 340 male) early neurological and neurosurgical rehabilitation patients have been analyzed. Mean age was 64.9 (15.4) years. Main diagnoses are displayed in Table 2. 36.5 (142.0) days since onset of neurological/neurosurgical disease had passed. Results of the scales on admission are displayed in Table 3. There were no significant differences of EFA scores (subscores and total) between stroke and head injury patients (*p* > 0.05).

**Fig. 1:**
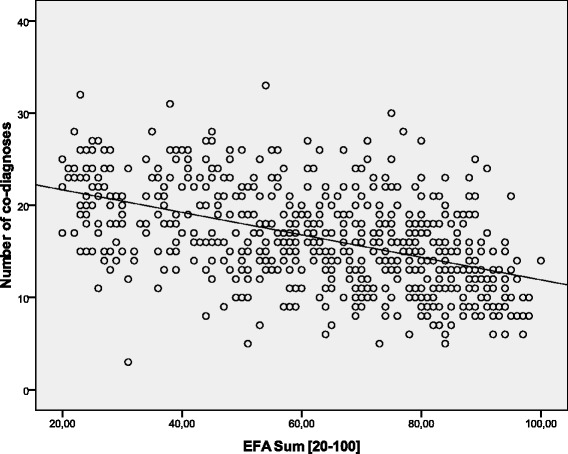
Correlation between EFA total score and number of co-diagnoses. The more co-diagnoses the smaller EFA scores were recorded

**Fig. 2:**
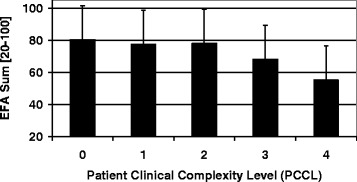
The higher PCCL, the lower the EFA total scores were observed. Mean EFA values (bars) and mean standard deviation is indicated (on top of bars)

**Fig. 3:**
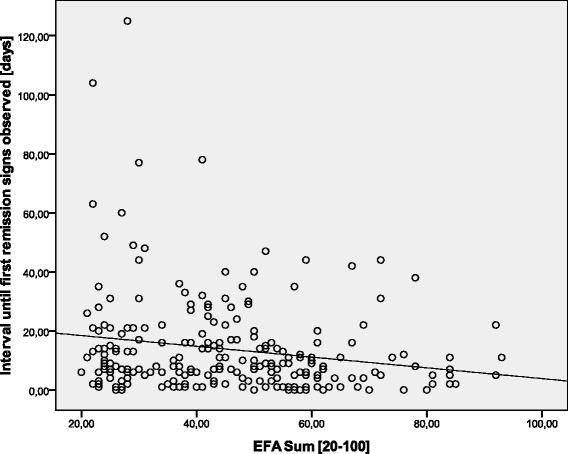
Interval until first remission signs were observed and EFA total score

**Fig. 4:**
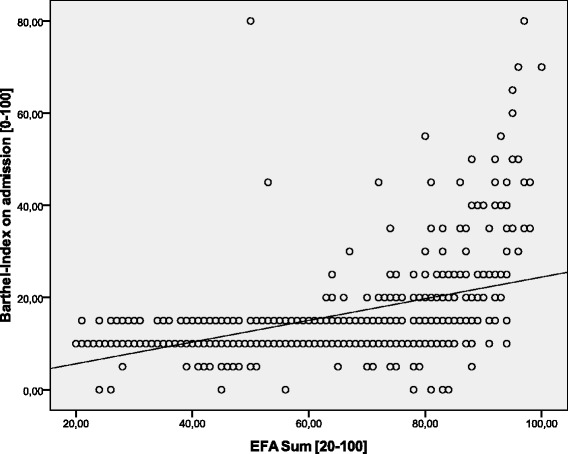
Correlation between EFA total score and BI on admission

**Fig. 5:**
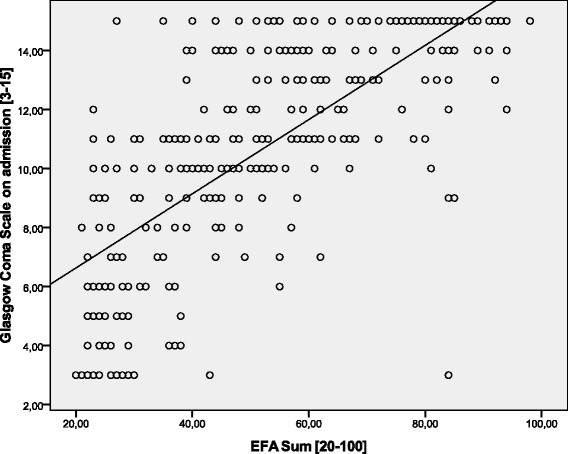
Correlation between GCS on admission and EFA total score

**Fig. 6:**
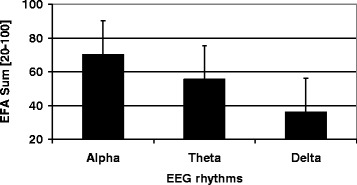
Mean EFA sums of patients with alpha, theta and delta EEG rhythms. Further, mean standard deviation is indicated on top of bars

**Fig. 7:**
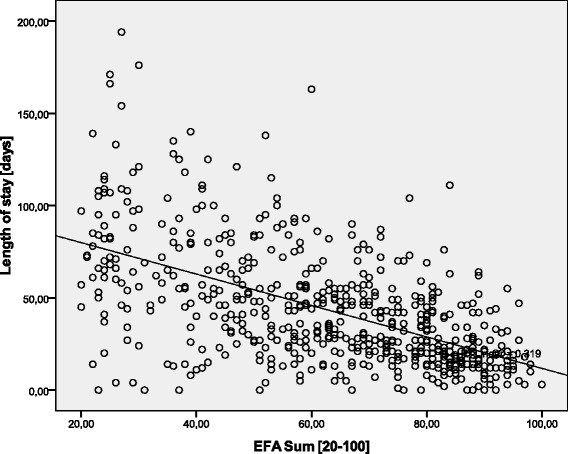
Correlation between length of stay (LOS) in neurological early rehabilitation and EFA total score

1.  Concurrent validity
  A mean of 16.4 (5.4) co-diagnoses per patient was documented. The more co-diagnoses, the lower EFA total scores were found (r_s_ = -0.509, *p* < 0.001, Fig. 1). 91.0 % of all patients (*n* = 567) had the highest Patient Clinical Complexity Level (PCCL) of 3 or 4. The higher the PCCL, the lower EFA total scores were found (Fig. 2). Patients with a PCCL of 4 had a significantly lower EFA total score than any other group (F = 22.82, all LSD-tests *p* < 0.001).
  For those patients with disorders of consciousness like coma or unresponsive wakefulness syndrome (*n* = 238), first remission signs were observed after a mean of 13.6 (16.4) days. The longer the period until remission signs were observed, the lower the EFA total r_s_ = -0.192, *p* < 0.01, Fig. 3), oro-facial (r_s_ = -0.179, *p* < 0.01), sensorimotor (r_s_ = -0.153, *p* < 0.05) and cognitive score on admission (r_s_ = -0.172, *p* < 0.01). There was, however, no significant correlation between remission time and EFA vegetative.
  Autonomic instability was observed for a mean of 25.8 (28.7) days after admission. EFA vegetative correlated significantly and negatively with duration of vegetative problems (r_s_ = -0.341, *p* < 0.001).
  EFA total score correlated significantly with BI (r_s_ = 0.570, *p* < 0.001, Fig. 4), ERI (r_s_ = 0.505, *p* < 0.001), CRS (r_s_ = 0.732, *p* < 0.001) and GCS (r_s_ = 0.751, *p* < 0.001, Fig. 5) on admission. EFA vegetative and cognitive abilities domains as a measure of wakefulness correlated significantly and positively with GCS (r_s_ = 0.560 resp. r_s_ = 0.727, *p* < 0.001). EFA oro-facial and cognitive as a measure of communication and facial motor abilities correlated with CRS subscale talking (r_s_ = 0.370 resp. r_s_ = 0.299, *p* < 0.001). Further, EFA cognitive was significantly lower when a disturbance of communication was documented in the ERI: 13.8 (5.8) vs. 18.4 (5.8), t = 8.3, *p* < 0.001. Patients with tracheostomy (indicating dysphagia and danger of aspiration) had a significantly lower EFA oro-facial score than patients without: 7.4 (4.1) vs. 15.7 (4.8), t = 18.6, p < 0.001. EFA sensorimotor abilities as a measure of mobility correlated with CRS subscale motor (r_s_ = 0.539, *p* < 0.001) and BI (r_s_ = 0.528, *p* < 0.001).
  EEG patterns of *n* = 183 patients were analyzed, Alpha rhythm (8-13 hz) was found in *n* = 124, theta (4-7 Hz) in *n* = 53 and delta (0.5-3 Hz) in only *n* = 6 patients. EFA total scores differed highly significantly between the three EEG rhythms (ANOVA, F = 19.8, *p* < 0.001). In post-hoc LSD-tests, EFA total scores were lower among patients with theta (*p* < 0.001) and delta (*p* < 0.001) than alpha rhythms. In addition, EFA scores of patients with delta and theta rhythms differed significantly (*p* < 0.05), Fig. 6.
2.  Prognostic validity
  Patients with poor outcome (BI at discharge < 50) had significantly lower EFA total, vegetative, oro-facial, sensorimotor and cognitive scores than patients with better outcome (*p* < 0.001), Table 4. BI at discharge correlated positively with EFA on admission (r_s_ = 0.571, *p* < 0.001). Mean length of stay (LOS) in early rehabilitation was 44.4 (38.2) days. EFA total score and LOS correlated negatively (r_s_ = -0.565, *p* < 0.001), Fig. 7.
  A univariate analysis of variance (ANOVA) was performed using the following model: BI at discharge as dependent variable; main diagnose, PCCL and EEG rhythm as categorical independent variables; ERI, BI, GCS and EFA sum on admission, age, number of co-diagnoses, remission time and duration of autonomic instability as independent covariates. This model explained 81.7 % of the data variation (*p* < 0.001). EFA sum (F = 28.0, *p* < 0.001), ERI (F = 10.5, *p* < 0.01), BI (F = 13.9, *p* = 0.001) and GCS (F = 5.4, *p* < 0.05) on admission turned out to have a significant influence on BI at discharge (outcome), only.

**Table 2:** Main diagnoses

Diagnosis	Number	Percent
Ischemic stroke	226	36.3
Cerebral hemorrhage (non-traumatic)	111	17.8
Subarachnoidal bleeding (non-traumatic)	65	10.4
Cerebral hypoxia	27	4.3
Tumor	22	3.5
Polyneuropathy/Guillain-Barre-syndrome	19	3.0
Head injury	18	2.9
Spinal trauma	13	2.1
Other main diagnosis	122	19.6
Sum	623	100

**Table 3:** Scores of the different scales on admission

Scale	Mean (standard deviation)
EFA vegetative [4 – 20]	11.1 (4.0)
EFA oro-facial [4 – 20]	13.8 (5.8)
EFA sensorimotor [7 – 35]	20.4 (8.5)
EFA cognitive function [5 – 25]	17.3 (6.1)
EFA sum [20 – 100]	63.4 (21.3)
Barthel Index (BI) [0 – 100]	16.2 (11.5)
Early Rehabilitation Index (ERI) [-325 – 0]	−52.3 (55.4)
Early Rehabilitation Barthel Index (ERBI) [-325 – 100]	−36.2 (58.7)
Glasgow Coma Scale (GCS) [3 – 15]	10.7 (3.7)
Coma Remission Scale (CRS) [0 – 24]	11.3 (6.7)

**Table 4:** EFA scores on admission and outcome at discharge

	Poor outcome (BI < 50 at discharge)	Good outcome (BI ≥ 50 at discharge)	t-value	*p*
EFA vegetative	10.1 (3.5)	12.9 (4.2)	−8.9	*p* < 0.001
EFA oro-facial	12.3 (5.9)	16.6 (4.6)	−9.1	*p* < 0.001
EFA sensorimotor	18.1 (7.8)	25.1 (7.9)	−10.5	*p* < 0.001
EFA cognitive	15.8 (6.2)	20.3 (4.5)	−9.3	*p* < 0.001
EFA total	56.1 (20.3)	75.0 (12.3)	−11.2	*p* < 0.001

## Discussion

The ERBI is frequently used in Germany to assess the progress of neurological and neurosurgical early rehabilitation patients [4]. However, the ERBI has some limitations. It focuses on activities of daily living (ADL) and some items relevant to this group of patients, such as tracheotomy or mechanical ventilation [4]. Wakefulness and cognitive items are missing in the ERBI. Well established scales like GCS [13] or CRS [14] measure wakefulness but do not comprise ADL or cognitive items.

The EFA assessment has been developed to evaluate cognitive abilities (including wakefulness) as well as items of ADL among neurosurgical early rehabilitation patients [24, 25]. While inter-rater reliability was found to be moderate to good [25], no studies on its validity are available, yet. The rationale of the present study was to contribute further knowledge to the question whether the EFA scale is a valid instrument to assess progress of early rehabilitation patients as well as its prognostic value.

While neurological early rehabilitation patients suffer from different disorders, no significant differences of EFA scores on admission could be detected when comparing stroke and head injury patients suggesting that EFA scale may be useful for a broad spectre of diagnoses. We found that EFA total scores correlated very well with measures of morbidity, such as PCCL or number of co-diagnoses. In addition, EFA on admission predicted LOS, remission time and duration of autonomic instability. These findings indicate that patients with higher morbidity had lower EFA values on admission. It seems reasonable to believe, that critically ill patients have worse functional abilities than healthier subjects.

EFA also predicted outcome. There was a highly significant correlation between EFA on admission and BI at discharge. In addition, patients with a poor outcome (BI < 50) had significantly lower EFA total and category scores. In an ANOVA, EFA sum had the highest significant influence on BI at discharge (*p* < 0.001).

Comparing EFA with well established scales like ERBI [4], BI, GCS or CRS [13, 14], there were also highly significant correlations. The higher EFA scores were observed, the higher GCS and CRS values could be obtained.

As a neurophysiologic measure of wakefulness, faster EEG rhythms could be observed among patients with higher EFA scores. This finding indicates that the EFA scale seems to be of some value when assessing patients` wakefulness.

Some limitations of the study need to be addressed. First of all, it has to be pointed out that it was a retrospective data analysis. In general, the quality of the database is better in prospective controlled studies. Secondly, the study contributes some knowledge to the concurrent and prognostic validity of the EFA scale, but inter-rater-reliability has not been examined at all. Only well experienced (in early rehabilitation) staff has been involved in the study, but rating of BI, ERI and EFA might differ considerably between different professions (e.g. nurses and physicians). Thirdly, more sophisticated statistical analyses like an Item Response Theory (IRT) analysis could be of some value.

## Conclusions

The concurrent and prognostic validity of the EFA scale is supported by the finding that it correlates with morbidity, LOS, established scales of wakefulness, ADL and outcome. The EFA scale may be used to evaluate progress of vegetative, oro-facial, sensorimotor and cognitive functions of critically ill neurological and neurosurgical early rehabilitation patients. Prospective and controlled studies on reliability, in particular inter-rater reliability, and validity of the EFA assessment are strongly encouraged.
